# Community-based randomized controlled trial of diabetes prevention study for high-risk individuals of type 2 diabetes: lifestyle intervention using web-based system

**DOI:** 10.1186/s12889-017-4283-y

**Published:** 2017-05-05

**Authors:** Seon-Ah Cha, Sun-Young Lim, Kook-Rye Kim, Eun-Young Lee, Borami Kang, Yoon-Hee Choi, Kun-Ho Yoon, Yu-Bae Ahn, Jin-Hee Lee, Seung-Hyun Ko

**Affiliations:** 1 Division of Endocrinology & Metabolism, Department of Internal Medicine, St. Vincent’s Hospital, College of Medicine, The Catholic University of Korea, Jungbu-daero 93, Paldal-gu, Suwon-si, Geyonggi-do 442-723 South Korea; 2 Catholic Institute of U-Healthcare, Institute of Biomedical Industry, The Catholic University of Korea, 222 Banpo-daero Seocho-gu, Seoul, 06591 South Korea

**Keywords:** Type 2 diabetes, Diabetes prevention, Lifestyle intervention

## Abstract

**Background:**

The trend of increasing numbers of patients with type 2 diabetes emphasizes the need for active screening of high-risk individuals and intensive lifestyle modification (LSM).

**Methods/design:**

The community-based Korean Diabetes Prevention Study (C-KDPS) is a randomized controlled clinical trial to prevent type 2 diabetes by intensive LSM using a web-based program. The two public healthcare centers in Korea are involved, and 420 subjects are being recruited for 6 months and will be followed up for 22 months. The participants are allocated randomly to intensive LSM (18 individual sessions for 24 weeks) and usual care (control group). The major goals of the C-KDPS lifestyle intervention program are: 1) a minimum of 5–7% loss of initial body weight in 6 months and maintenance of this weight loss, 2) increased physical activity (≥ 150 min/week of moderate intensity activity), 3) balanced healthy eating, and 4) quitting smoking and alcohol with stress management. The web-based program includes education contents, video files, visit schedules, and inter-communicable keeping track sites. Primary outcomes are the diagnoses of newly developed diabetes. A 75-g oral glucose tolerance test with hemoglobin A1c level determination and cardiovascular risk factor assessment is scheduled at 6, 12, 18, and 22 months.

**Discussion:**

Active screening of high-risk individuals and an effective LSM program are an essential prerequisite for successful diabetes prevention. We hope that our C-KDPS program can reduce the incidence of newly developed type 2 diabetes and be implemented throughout the country, merging community-based public healthcare resources and a web-based system.

**Trial registration:**

Clinical Research Information Service (CRIS), Republic of Korea (No. KCT0001981). Date of registration; July 28, 2016.

## Background

Type 2 diabetes, with its accompanying complications, is an enormous health burden worldwide. The cost related to diabetes was U.S. $245 billion in 2012 in the U.S., and U.S. $ 673 billion in 2015, representing about 11% of worldwide healthcare expenditure [1, 2]. In 2013, total antidiabetic pharmacy expenditure was U.S. $4 billion (480 billion won) in Korea [3]. Moreover, the higher prevalence of prediabetes than of diabetes is a more serious health concern that requires urgent intervention. Diabetes prevention should be the first step.

Preventive effects of lifestyle intervention (LSI) with diet and exercise have been proven in adults with high risk of type 2 diabetes in several landmark randomized clinical trials (RCTs). [4]. The first RCT of LSI, the Finnish Diabetes Prevention Study (DPS), showed a 58% relative reduction in the incidence of diabetes compared to the control group [5]. In addition, sustained lifestyle changes and a reduction in type 2 diabetes incidence after 3 years of stopping LSI have been demonstrated [6]. The LSI program of the U.S. Diabetes Prevention Program (DPP) decreased the incidence of new type 2 diabetes by 58%, compared with 31% in a metformin-treated group [7]. Even during the 10 years’ follow-up since randomization to U.S. DPP (DPP Outcome Study), the diabetes incidence rate was reduced by 34% in the lifestyle group compared with placebo [8]. In the Da-Qing study, involving 577 Chinese people with impaired glucose tolerance, the risk of type 2 diabetes was reduced by 31% with diet, 46% with exercise, and 42% with diet plus exercise [9]. Therefore, LSI is a powerful and long-term strategy to prevent type 2 diabetes.

However, a diabetes prevention trial conducted in a community is inadequate. The Reaching Out to Prevent Increases in Diabetes (RAPID) study was a randomized effectiveness trial designed to evaluate the weight loss effectiveness of a YMCA model for the DPP intervention [10]. This YMCA model for translating the DPP intervention achieved significant weight loss at 12 months among low-income adults [10]. In addition, several countries tried to implement type 2 diabetes prevention actions in public health or clinical care [11–15]. These studies have shown that diabetes is preventable, and population-level approaches are needed for widespread implementation [16]. To do this, a careful and thorough review is needed for selection of target population and resources; establishment of appropriate target goals; determination of intervention modality, mode of delivery, and feasibility of the intervention program; and consideration of cultural differences, and so on [16, 17].

Korea has a national health screening examination service, provided by the National Health Insurance Service (NHIS) through 254 public healthcare centers across the country, and an excellent internet infrastructure. Korean people aged 40 years and older are required to undergo a mandatory health examination survey every 2 years, and the costs are covered by the NHIS in Korea [18]. In addition, the internet penetration rate of Korea is 95%, and the internet speed ranks as the first in the world. Even elderly people can use the internet and mobile devices with or without help. Therefore, the conditions are favorable both for the screening of high-risk individuals for type 2 diabetes and for internet-based LSI in Korea.

Currently, Korea has no available diabetes prevention program operated by an internet-based LSI system in the community setting, especially not in public healthcare centers run by the government. To the best of our knowledge, this is the first RCT study of a translational diabetes prevention program using a web-based system in a community setting.

## Aims

The purpose of this manuscript is to provide a detailed description of the web-based LSI program used in a community setting in the Korean Diabetes Prevention Study (KDPS).

## Methods

### C-KDPS steering committee

The community-based KDPS (C-KDPS), is an intensive LSI program that was developed by the C-KDPS steering committee from the two Diabetes Centers of the Catholic University of Korea (Seoul St. Mary’s Hospital and St. Vincent’s Hospital) and the Institute of U-Healthcare core in December 2015. The committee members are composed of the principal investigator from each clinical center, a diabetologist, a diabetes educator, a registered dietitian, exercise physiologists, and a web programmer. The C-KDPS program was based on the U.S. Diabetes Prevention Program (DPP). After an extensive literature review, the committee members developed the C-KDPS study protocol on the basis of recommendations from the SPIRIT statement on randomized trials (Table 1) [19]. The principal investigator of each center plays a role in the communications between the committee and two public healthcare centers in Chungju City and Suwon City in Korea. The committee members held regular meetings to check the process of preparing, tracking, and updating the progress on implementing the C-KDPS program in the two cities every week from December 2015. The lifestyle modification (LSM) protocol and development process was reviewed and approved by IRBs of St. Vincent’s hospital (No. VC16MISI0003) and Seoul St. Mary’s hospital (No. KC16EISI0234). This trial has been registered with the Clinical Research Information Service (CRIS), Republic of Korea (KCT0001981). This C-KDPS program protocol has also been reviewed and certified by the Korean Diabetes Association.

**Table 1:** Proposed timeline of C-KDPS

Year	2016		2017				2018				2019
Term	3	4	1	2	3	4	1	2	3	4	1
Enrollment											
1^st^ screening	X	X									
2^nd^ screening		X	X								
Informed consent		X	X								
Allocation		X	X								
Intervention											
LSI		X	X	X	X	X	X	X	X	X	
Assessment											
6-month FU				X	X						
12-month FU						X	X				
18-month FU								X	X		
22-month FU										X	X
Web system											X

*FU* follow-up

### Study design

**Fig. 1:**
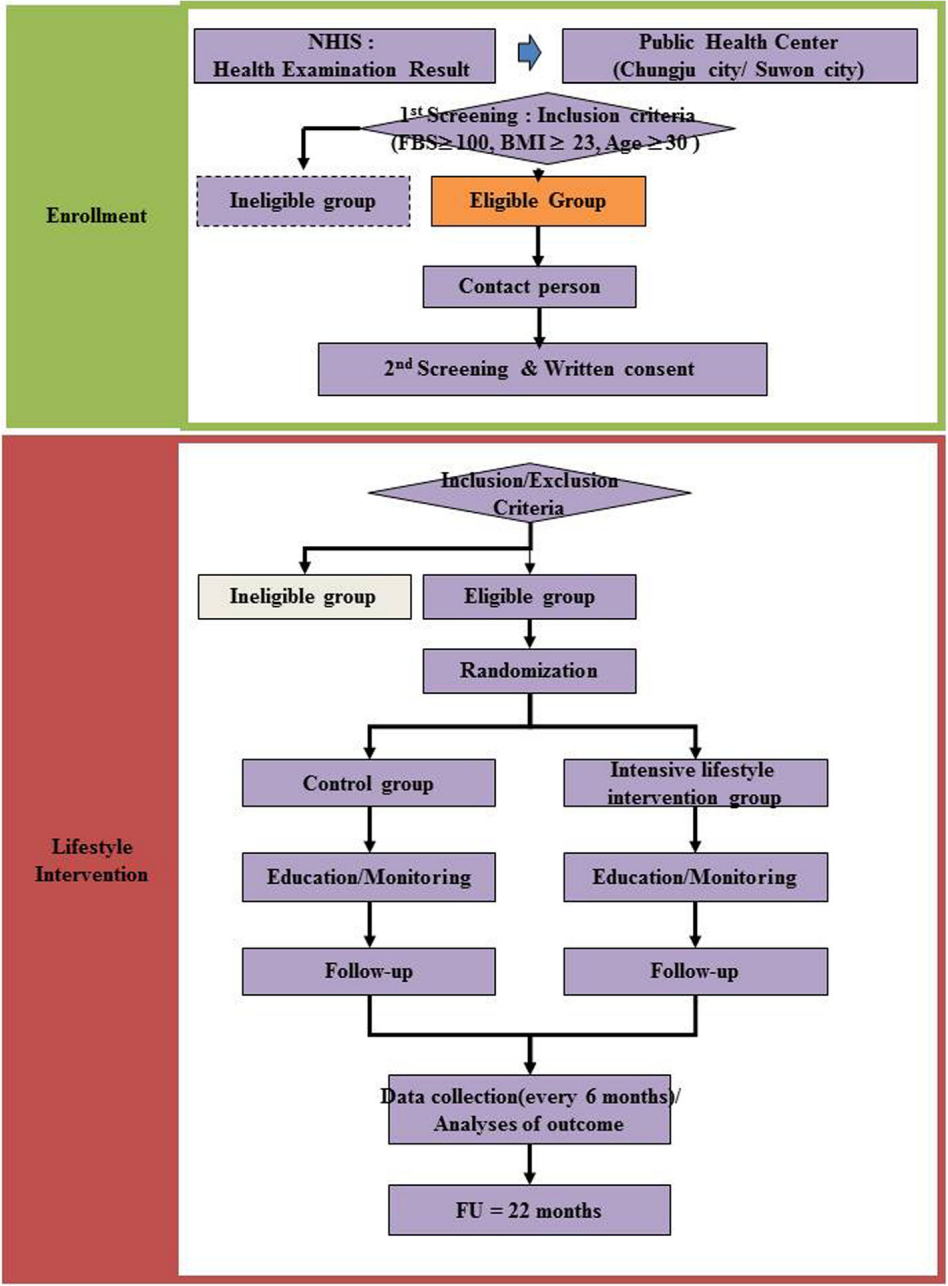
Schematic diagram of study protocol

The Community-Based Korean Diabetes Prevention Study (C-KDPS) is a prospective RCT. A total of 420 subjects at high risk of developing type 2 diabetes will be enrolled in this study. The participants are randomly assigned either to a control group or to an intensive LSI group. Figure 1 shows the flow of participants through the study in each center.

### Randomization

In this study, a stratified blocked randomization strategy will be used to achieve between-group comparability, and to avoid imbalance in the number of subjects in each stratum. The randomization entails measuring the level of the stratified factors—center and sex—for eligible subjects, determining to which stratum each belongs, and using a block size of 4 within each stratum. Stratified blocked randomization in both centers will be conducted by study staff not involved in the intervention through a central computerized process. The sequence of group allocations will be concealed until after the baseline data collection is completed.

### Recruitment

Eligible individuals should be in the pre-diabetic range of diabetes: fasting plasma glucose (FPG) 100–125 mg/dL, 2-h post-load plasma glucose (PG) 140–199 mg/dL, or HbA1c 5.7–6.4% (39-46 mmol/mol) [20]. The age and body mass index (BMI) criteria are set at 30 years and older and ≥23 kg/m^2^.

Exclusion criteria are as follows: 1) previous diagnosis of diabetes or diabetes treatment; 2) inability to adhere to lifestyle modification; 3) uncontrolled hypertension or significant ischemic heart disease requiring hospitalization within 6 months of enrollment; 4) either heart failure, anemia (WHO criteria), or cancer requiring treatment in the past 5 years or renal insufficiency; 5) severe liver dysfunction or alcohol abuse; 6) significant arthritis or psychiatric problems; 7) either pregnant or planning to become pregnant; 8) several medications (thiazide, beta blockers, steroid, immune-modulating agents, agents for weight reduction); and 9) other systemic illness affecting the performance of this intervention program.

The screening process is a two-step approach. If the examinees of a national health examination agree to provide their personal information, informed consent is obtained at the time of the health examination. The “healthcare manager” in healthcare centers in Chungju and Suwon city can gain access to the database consisting of the health examination results of the examinees of the habitable areas covered by the healthcare centers. The candidates of this study who are aged ≥30 years, with a BMI ≥ 23 kg/m^2^ are initially screened by fasting glucose level (100-125 mg/dL) from the NHIS database. The C-KDPS program is announced to candidates in both Chungju and Suwon city via brochure or telephone, banner exhibition in the public healthcare centers, and extensive advertisement through the healthcare centers’ web pages.

After initial screening, the participants are instructed to visit their nearby public healthcare centers after overnight fasting. With the informed consent process, the subjects perform a 75-g oral glucose tolerance test (OGTT), HbA1c level, and questionnaires regarding lifestyle habits. Additional blood sampling is done at 30 and 120 min for PG, insulin, and C-peptide levels using the standardized manual of operations [21], and shipped to the central laboratory within 24 h of sample collection. The questionnaires contain self-reported personal data about medical history, employment, education, economic status, smoking or alcohol use, and lifestyle habits including diet and physical activity habits. If the glucose and HbA1c levels of subjects are within the pre-diabetic range, the subjects are recommended to participate in this C-KDPS. In addition, demographic data and a standard 12-lead electrocardiogram are obtained. Anthropometric and laboratory analysis are scheduled to be conducted at baseline, 6, 12, 18, and 22 months.

Recruitment of participants began on July 1, 2016. The enrollment of the participants is expected to be complete by the end of January 2017. Intervention and follow-up of study subjects will be ongoing until December 2018.

### Control arm

After randomization, subjects allocated to the control group will receive one 30-min individual session, provided by a coordinator, about the importance of healthy eating and physical activity to prevent type 2 diabetes [4, 22]. The toolkit, including leaflets about healthy eating and instructions for physical activity, is presented to participants in control group. They are instructed to visit a public healthcare center for follow-up, and no additional education is provided.

### Intensive lifestyle intervention (LSI) core program

The LSI protocol was developed based on the U.S. DPP and a previous literature review related to community-based intervention studies [4, 22]. The major goals of the C-KDPS LSI program are: 1) a minimum of 5–7% loss of initial body weight in 6 months and to maintain this weight loss throughout the study period, 2) increased physical activity of at least 150 min/week with moderate-intensity activity, 3) balanced healthy eating habit, and 4) cessation of smoking and alcohol with stress management. The intensive LSI program is a systematically organized system using web-based program, designed to motivate, empower, and encourage the participants for long-term adherence [23]. Trained C-KDPS coordinators (background in nursing) deliver all the intervention sessions using a web-based system.

The LSI program curriculum is delivered in 18 sessions over 24 weeks (Table 2). During the first 12 weeks, all participants are instructed to visit every week. After one session of orientation, instructions for modifying lifestyle, dietary intake, physical activity, and self-monitoring are introduced for all 18 sessions. During latter 12 weeks, the program focuses on adherence to the LSM. After the 24-week period, personal contact and monitoring are scheduled for at least every 2 months at the public healthcare centers to check adherence to the LSM. The coordinators have the primary responsibility for delivering the LSM program, conducting maintenance sessions, motivating the participants to keep their LSM, assessing the attainment of the goal, and acquisition of required data. All the coordinators are registered nurses, and C-KDPS manuals are provided to minimize the differences among the educators and standardize the intervention contents.

**Table 2:** Comparison of core curriculum between DPP and C-KDPS

Session	DPP	C-KDPS
1	Welcome to the lifestyle balance program	Welcome to the C-KDPS program
2	Be a fat detective	How to exercise?
3	Three ways to eat less fat	Reason to lose body weight
4	Healthy eating	Right walking
5	Move those muscles	Healthy eating 1
6	Being activity	Aerobic exercise
7	Tip the calorie balance	Healthy easting 2
8	Take charge of what’s around you	Stretching exercise
9	Problem solving	Food exchange
10.	The four keys to healthy eating out	Aerobic exercise
11	Talk back to negative thoughts	Food exchange
12	The slippery slope of lifestyle change	Whole body Resistance exercise
13	Jump start your activity plan	Elastic band exercise
14	Make social cues work for you	Eat out healthy
15	You can manage stress	Lower body strengthening exercise
16	Ways to stay motivated	Eating for hypertension, hyperlipidemia
17		Core exercise
18		Giving up drinking and no smoking

### Web-based program construction and its use

**Fig. 2:**
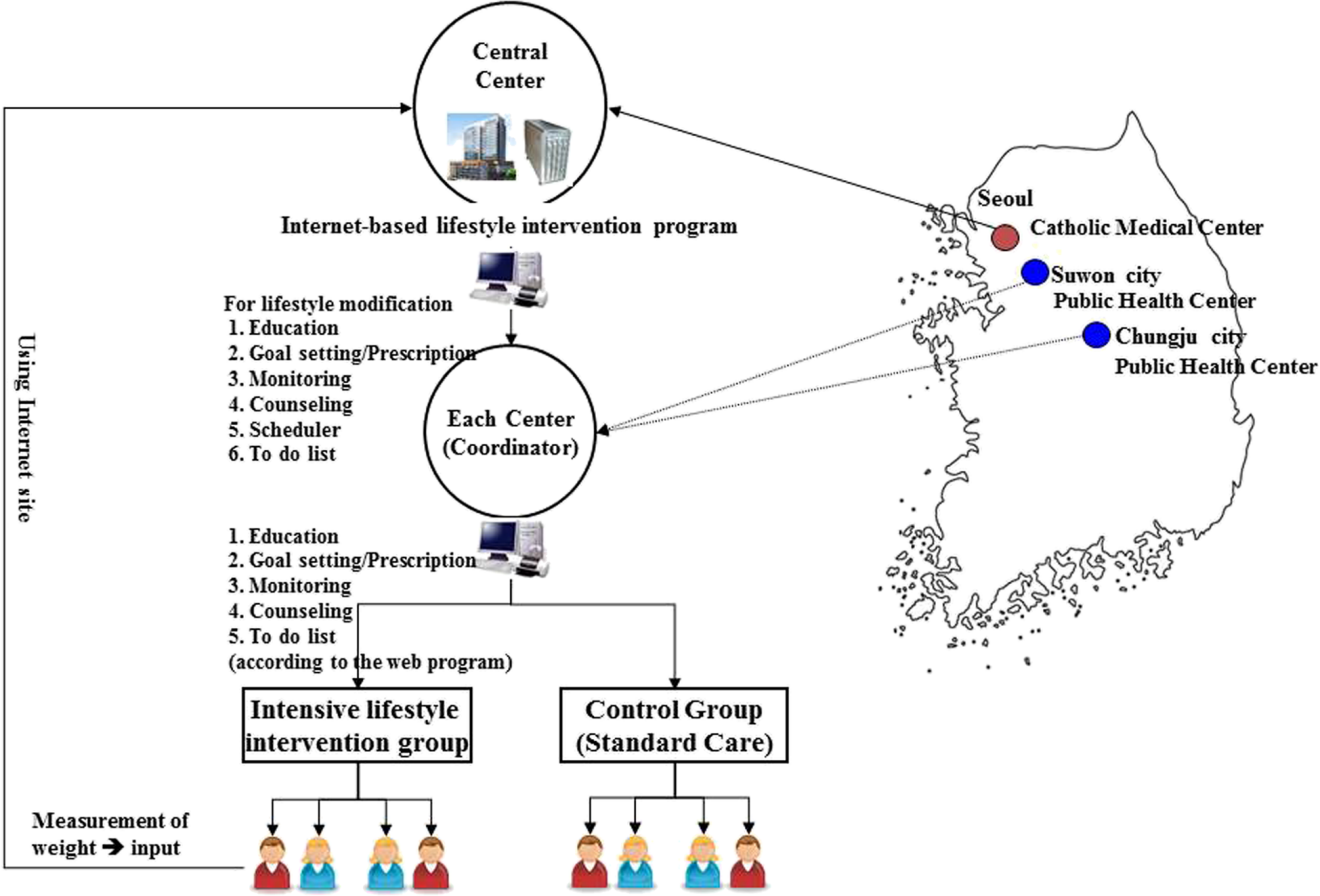
Study outline of this C-KDPS. Two healthcare centers in Chungju city and Suwon City were selected. The study was controlled and monitored by the Catholic Medical center in Seoul, Korea. A lifestyle intervention (LSI) program was performed through an internet-based web program that included education contents, goal setting and prescription, monitoring, counseling, and a scheduler. The principal investigator, the steering committee, and the coordinators (educators) communicated with each other in real time via the web site

The web site was designed to coordinate the C-KDPS process. It is composed of four parts: 1) personal information; 2) LSM program implementation (instruction, goal setting, education materials, case-report form, and recommendations); 3) mutual communication among participants, health coordinators, and committees (counseling, Q&A, free contact to coordinators); and 4) planner function (things to do, visit schedule) (Fig. 2).

As body weight change is a main parameter of each visit, the target goals for the next visit (body weight, exercise time, and dietary calorie) are automatically calculated, based on current body weight. The 7% weight loss goal is presented, and participants are encouraged to achieve this target. If the participants achieve the target body weight, maintenance of the weight is encouraged. If the participants lose more than 7% of initial body weight, they are recommended to maintain the weight that they have lost, as long as their BMI exceeds 21 kg/m^2^ [4]. During the core program, the participants visit the healthcare centers for 24 weeks, and join in supervised physical activity at every visit. The coordinators teach the exercise skills using video on the web site. The video file is available during the study period at any convenient time and place for participants. Supporting materials, such as pedometer (Mi Band, Xiaomi Inc., China), toolkit, exercise band, and hand grips are given after a supervised and corresponding class. The participants are instructed in the use of the equipment at the healthcare center and then take the equipment home. The fat and calorie goals are used to achieve the weight loss goal, not as goals for healthy eating. Dietary modification is set to achieve total daily calorie intake, and the calorie goals are calculated automatically, based on current body weight [4, 9].

Participants are instructed to use a “keeping track booklet” to record their food intake and adherence to physical activity every week for 24 weeks. At every visit, the subjects bring their booklets and review the contents with coordinator. The coordinator reinforces and encourages the subjects’ diet and exercise habits, while also measuring adherence to the LSI. If the participant fails to visit the public healthcare center, the coordinator tries to make contact by cell phone or text message. Subjects’ self-reported exercise and dietary habits are collected, as are data that they enter on the web site. Frequency and duration of physical activity are estimated on a weekly basis using questionnaires. Dietary intake is measured using a simple food frequency questionnaire. This process takes about 40–60 min every visit. The coordinators provide the LSM prescriptions and discuss participants with detailed plans.

### Maintenance strategy

After completion of the core curriculum for 24 weeks, the participants are instructed to visit every month for the next 6 months, and every 2 months thereafter, until the end of the study (Table 3). If they cannot visit the centers, the coordinator contacts them by phone and via the web site. As retention of participants throughout the study period is very important, a healthy lifestyle camp, group teaching, or a reinforcement program is scheduled for at least two classes per year to deliver messages about healthy eating, maintenance of weight goal, behavioral topics, and motivational programs.

**Table 3:** Frequency of management for each group while undertaking the prevention program

Intensive lifestyle intervention group			Control group	
subject prescription, education, monitoring		Management frequency	subject education	Management frequency
			(Only prescription)	
1-12 weeks	1/ week	12	1-12 weeks	1
13-24 weeks	1/2 weeks	6	13-24 weeks	1
25-48 weeks	1/month	6	25-48 weeks	1
1-2 years	2/month	5	1-2 years	1

### Training of coordinator and investigators

All coordinators are obliged to attend a biannual 1-day training session. At the initiation meeting, coordinators are instructed in the C-KDPS protocol, all intervention manuals, interview skills, and how to use the web site. After 6 months, the training program is scheduled to review LSI data, discuss the education process, role playing, maintenance skills, and feedback of the feasibility about the web-based program. The steering committee holds regular meetings with investigators and staff from two centers at least 2 times per month to review and check the study process.

### Study outcomes

The primary outcome is new development of diabetes diagnosed by 2016 ADA criteria. A 75-g OGTT with HbA1c level testing is scheduled at 6, 12, 18, and 22 months. Secondary outcomes consist of cardiovascular disease (CVD) risk factors, changes in demographic characteristics with pancreatic β-cell function, insulin sensitivity, lifestyle habits, quality of life, feasibility or compliance of bidirectional web-based program, and health costs for the C-KDPS.

### Statistical analysis

The C-KDPS sample size was determined for the primary outcome (between-group difference in diabetes incidence) at 22 months’ follow-up with type 1 error(α) = 0.05 and power (1-β) ≥ 0.80. We aimed to detect a cumulative incidence difference of 11% between the two intervention groups (control group = 19.8% vs. intensive LSI group = 8.6%) based on previous studies [7, 9]. A total of 420 subjects should be recruited, considering the dropout rate of 25% during the study period.

The primary and secondary outcomes will be analyzed using the intention-to-treat approach. Differences in participants’ baseline characteristics and clinical outcomes between two groups were evaluated by a two-sample *t*-test for continuous outcomes and by a Chi-square test or Fisher’s exact test for categorical outcomes. A mixed-model analysis of covariance, including treatment group, gender, age, and the terms and baseline values of clinical parameters, will be used to assess the effects of intervention in a post-intervention survey. For the primary outcome—the comparison of cumulative incidence of diabetes between two groups—a log-rank test with random effects for intercept to account for pre-post correlations within individuals will be used. An interaction effect between time and intervention group will be assessed first. If there is no interaction effect, the overall intervention difference will be assessed. Otherwise, the intervention difference will be assessed at each time point. Adherence to the LSI program is measured by evaluating class attendance, weight loss goal at 6 months, maintenance of physical activity, and achievement of diet goal. For long-term effects of intervention, cost-effectiveness analysis will be analyzed using the Markov model [24].

## Discussion

### Importance of diabetes prevention: To whom and how?

Type 2 diabetes is a serious health concern all over the world. In particular, people originating from South Asia and China develop type 2 diabetes at a higher rate, at an earlier age, and at a lower range of BMI than do their white counterparts [25]. Korea is no exception. The socioeconomic and health costs for diabetes are also enormous in Korea [26]. According to the annual report from the NHIS under the Ministry of Health and Welfare in Korea, diabetes is the sixth leading cause of death in the country and the fourth greatest expense in the health insurance budget [27]. Many countries take preventive measures at the government level. Such measures should be accompanied by an enormous budget, related professional manpower, and effective strategy. Therefore, selection of an appropriate target population and a cost-effective prevention program are needed.

A meta-analysis of RCTs demonstrated that intensive LSIs are highly effective in the Asian population, as elsewhere [28]. A meta-analysis of eight RCTs found a 45% reduction in the incidence of new type 2 diabetes in Asian people assigned to the intervention arm (OR 0.55, 95% CI 0.44–0.70) [29]. Almost all the diabetes prevention studies included subjects with pre-diabetes range. We designed the inclusion criteria for C-KDPS according to ADA criteria, and subjects with an HbA1c level of 5.7–6.4% were included as high-risk individuals.

However, even a perfect diabetes prevention program is of no use if the high-risk individuals are not screened or are inappropriately selected. Community-based screening of type 2 diabetes across the country is realistically impossible without sufficient budget. Therefore, community-based DPS could be tried if the healthcare system is provided by government in real practice. From this perspective, Korea has perfect conditions to perform a diabetes prevention program due to government-supported regular health examination service for all Korean people and public healthcare centers throughout the country. Public healthcare centers located near to the participant’s resident area would be the best places for C-KDPS.

### Implementation of DPP into community using internet-based monitoring program

This is the first RCT study of a translational diabetes prevention program using a web-based system in the community. Many previous reports and RCTs have revealed that LSIs aimed at reducing body weight and increasing physical activity helped to prevent diabetes in high-risk individuals with type 2 diabetes [22]. The key features of the methods to achieve lifestyle goals in DPP were a goal-based behavioral intervention; individual “lifestyle coaches” to deliver the intervention; frequent and regular contact throughout the trial; providing an individualized “toolbox” of adherence strategies; materials and strategies to address the needs of ethnic diversity; and an extensive local and national network of training and feedback with clinical support for the intervention [4]. The role of healthcare professionals should be as follows: delivering the correct information about the intensive LSM, monitoring participants’ LSM adherence, and encouraging participants. This is an ideal approach to maintaining of behavioral change, but it is very expensive. This hurdle could be partially overcome by use of information technology.

A web-based LSI program has many advantages. Standardized education contents, using slides, video images, manuals, and instructions, can be included without limit. Such a program reduces the social and health costs for training professional personnel in the field of dietetics and physical activity. It is possible to operate a high-quality intervention program independent of variance in coordinator quality. The number of subject visits can be reduced by use of a communicable web site. The education materials can be updated frequently. All of these strengths contribute to reducing health costs. Therefore, if the structured intervention program is provided using manuals or a web-based system, DPP can be implemented in the community with a low cost and standardized system.

The limitation of this study design is that metformin treatment is not included in the study arm. In this community setting, physician should prescribe the medication (metformin) and check regular laboratory monitoring about the safety of metformin use. However, physicians are not allocated in all of the public healthcare centers in Korea.

In conclusion, two key points, the screening of high-risk individuals and an effective LSI program, are essential for successful diabetes prevention. National health screening examination for all Korean people is established, public healthcare centers are easily accessible, and the country has an excellent internet infrastructure. We hope that our C-KDPS program might reduce the incidence of newly developed type 2 diabetes throughout the country by being implemented within a community-based healthcare system. Support from policymakers and government is very important for translational implementation of LSI programs to the community and nationwide level. Our study is expected to contribute to the launch of a health promotion program to prevent type 2 diabetes by merging public health resources and a web-based system. In future, long-term maintenance and economic aspects of this system should be evaluated. The cost and sustainability of an LSI program will make or break such a program.

## Abbreviations

BMI: Body mass index
CRIS: Clinical Research Information Service
CVD: Cardiovascular disease
DPP: Diabetes prevention program
DPS: Diabetes Prevention Study
FPG: Fasting plasma glucose
KDPS: Korean Diabetes Prevention Study
LSI: Lifestyle intervention
LSM: Lifestyle modification
NHIS: National Health Insurance Service
OGTT: Oral glucose tolerance test
PG: Plasma glucose
RAPID: Reaching Out to Prevent Increases in Diabetes
RCT: Randomized controlled trial
